# mHealth intervention to improve quality of life in patients with chronic diseases during the COVID-19 crisis in Paraguay: A study protocol for a randomized controlled trial

**DOI:** 10.1371/journal.pone.0273290

**Published:** 2022-11-08

**Authors:** Tamara Escrivá-Martínez, Mª Dolores Vara, Nadia Czeraniuk, Matías Denis, Francisco J. Núñez-Benjumea, Luis Fernández-Luque, Alba Jiménez-Díaz, Vicente Traver, Juan José Llull, Antonio Martínez-Millana, Jorge Garcés-Ferrer, Marta Miragall, Rocío Herrero, Analía Enríquez, Verena Schaefer, Sergio Cervera-Torres, Cecilia Villasanti, Carmen V. Cabral, Irene Fernández, Rosa Mª Baños

**Affiliations:** 1 Polibienestar Research Institute, University of Valencia, Valencia, Spain; 2 CIBER-Obn Physiopathology of Obesity and Nutrition, Instituto de Salud Carlos III, Madrid, Spain; 3 Centro de Investigación y Documentación, Universidad Autónoma de Encarnación, Encarnación, Paraguay; 4 Adhera Health, Inc, Palo Alto, CA, United States of America; 5 Department of Personality, Evaluation and Psychological Treatment, Faculty of Psychology, University of Valencia, Valencia, Spain; 6 ITACA Institute, Polytechnic University of Valencia, Valencia, Spain; 7 Department of Psychology and Sociology, Faculty of Social and Human Sciences, University of Zaragoza, Teruel, Spain; 8 Instituto Superior de Educación Divina Esperanza, Encarnación, Paraguay; 9 Hospital Regional de Encarnación, Encarnación, Paraguay; 10 Department of Methodology for the Behavioral Sciences, Faculty of Psychology, University of Valencia, Valencia, Spain; Prince Sattam Bin Abdulaziz University, College of Applied Medical Sciences, SAUDI ARABIA

## Abstract

**Background:**

Patients with chronic disease represent an at-risk group in the face of the COVID-19 crisis as they need to regularly monitor their lifestyle and emotional management. Coping with the illness becomes a challenge due to supply problems and lack of access to health care facilities. It is expected these limitations, along with lockdown and social distancing measures, have affected the routine disease management of these patients, being more pronounced in low- and middle-income countries with a flawed health care system.

**Objectives:**

The purpose of this study is to describe a protocol for a randomized controlled trial to test the efficacy of the Adhera® MejoraCare Digital Program, an mHealth intervention aimed at improving the quality of life of patients with chronic diseases during the COVID-19 outbreak in Paraguay.

**Method:**

A two-arm randomized controlled trial will be carried out, with repeated measures (baseline, 1-month, 3-month, 6-month, and 12-month) under two conditions: Adhera® MejoraCare Digital Program or waiting list. The primary outcome is a change in the quality of life on the EuroQol 5-Dimensions 3-Levels Questionnaire (EQ-5D-3L). Other secondary outcomes, as the effect on anxiety and health empowerment, will be considered. All participants must be 18 years of age or older and meet the criteria for chronic disease. A total of 96 participants will be recruited (48 per arm).

**Conclusions:**

It is expected that the Adhera® MejoraCare Digital Program will show significant improvements in quality of life and emotional distress compared to the waiting list condition. Additionally, it is hypothesized that this intervention will be positively evaluated by the participants in terms of usability and satisfaction. The findings will provide new insights into the viability and efficacy of mHealth solutions for chronic disease management in developing countries and in times of pandemic.

**Trial registration:**

ClinicalTrials.gov NCT04659746.

## Introduction

The arrival of the COVID-19 has significantly impacted people’s mental health worldwide [1]. Evidence has shown that feelings of fear, uncertainty and loneliness, and symptoms of stress, anxiety and depression, are very prevalent in most individuals after the COVID-19 outbreak [2]. In Paraguay’s context, infection cases increased rapidly as in the rest of the world [3]. The study conducted by Torales et al. [4] showed that most of the Paraguayan population showed a moderate level of self-perceived stress, particularly in women, singles, and those who reported a pre-existing mental disorder such as depression or anxiety.

People with chronic disease (CD) (e.g., hypertension, diabetes, cardiovascular disease, cancer) are one of the groups most vulnerable to COVID-19 infection [5] and associated mental health problems [6]. Moreover, the implementation of the lockdown measures has impacted these people’s lifestyle [7, 8]. The current health crisis has exacerbated difficulties in accessing medical services and treatment, impacting these diseases’ clinical course [9]. Thus, there is a need to develop and disseminate innovative online care tools to engage at-risk populations in effective CD management during the COVID-19 era.

Throughout this pandemic, the integration of digital health solutions (e.g., smartphones) into CD management has improved access to quality and affordable care for patients by facilitating treatment adherence and behavioral changes [10]. Considering the increasing use and access to the Internet and mobile technology by the Latin American population, digital health could help overcome the healthcare gap in this population [11]. However, it remains challenging to address existing barriers to implementing and disseminating effective mHealth initiatives to improve the most vulnerable communities’ physical and mental health in a low-resource context [12].

Scientific literature suggests that mHealth solutions, including mobile applications (apps), help manage a CD [13, 14], including its possible comorbidity with other psychological problems [15]. In particular, the changes in CD patients’ lifestyle through the use of the mHealth apps are remarkable, mainly in adherence to regular exercise, healthy diet and body weight [16]. Besides, a growing body of research examines apps’ possibilities to improve quality of life or well-being in CD patients through different theoretical approaches (e.g., positive psychology), yielding promising results [17, 18].

Despite the wide variety of apps available for the management of CD during the COVID-19 situation, their quality and efficacy are generally unknown [10, 19]. Therefore, more studies are needed to rigorously ensure the efficacy, efficiency and cost-effectiveness of mHealth solutions. In this regard, this paper aims to describe a protocol for a randomized controlled trial (RCT) to test the efficacy of the Adhera® MejoraCare Digital Program to improve the quality of life of CD patients during the COVID-19 situation in Paraguay, comparing to the waiting list (WL) condition. Adhera® MejoraCare Digital Program (Adhera Health, Inc., Palo Alto, CA) is an mHealth intervention focused on tailored education, behavior change and emotional well-being. We hypothesize that a) Adhera® MejoraCare Digital Program will improve quality of life compared to WL condition 1 month after baseline, and this result will be maintained over time (3-, 6- and 12-month follow-ups); b) Adhera® MejoraCare Digital Program will show significant improvements in emotional distress (anxiety, depression and stress) 1 month after baseline compared to the WL condition, and these results will be maintained over time (3-, 6- and 12-month follow-ups); c) participants in this intervention will positively value the mHealth solution in terms of usability and satisfaction; and d) variables such as self-efficacy and health empowerment will show significant relationships with the change in the quality of life outcome, being significant predictors.

## Material and methods

### Research design

A two-arm RCT will be carried out with repeated measures (baseline, 1-, 3-, 6-, and 12-month follow-ups) and two conditions: Adhera® MejoraCare Digital Program and WL group. The study will be conducted following the CONSORT statement (Consolidated Standards of Reporting Trials, http://www.consort-statement.org) [20] and CONSORT-EHEALTH guidelines [21] and the Recommendations for Interventional Trials (SPIRIT) [22, 23].

### Sample size

Sample size calculation was done using G*Power [24], based on the primary endpoint. Specifically, sample size was calculated for a mixed ANOVA design, with the between effects being the experimental condition (2 groups) and the within-effects being the different time points. As we were interested in the interaction between experimental group and time effect, 8 measurements were specified (2 groups x 4 time points). With a power of 80%, and an alpha level = .05, and an expected moderated effect size of d = .50 according to similar studies [25], the required sample size is 74 participants (37 per condition). Taking into account a potential dropout of 20% according to literature [26, 27], this study will recruit a total of 94 participants (47 per condition).

### Study population, recruitment, and eligibility criteria

The study will be carried out in the region of Itapúa (Paraguay), in collaboration with the Regional Hospital of Encarnación, where the Autonomous University of Encarnación has its Research and Documentation Center.

The department of Itapúa is located in the extreme south of Paraguay, and its capital is the city of Encarnación. According to the last national census [28], the population of the department of Itapúa is 625.096 inhabitants, which represents 8.5% of the total population of the country. Specifically, 85% of the area of the Encarnación district is rural. Regarding education, illiteracy rate is 7%, and there is a difference between the illiteracy rate between the rural and urban population of 18% and the average year of schooling is 8 years. Regarding the use of digital devices, technology is increasingly being incorporated into the lives of Paraguayans. Specifically, 65% of people use the Internet in Itapúa [29].

**Fig 1:**
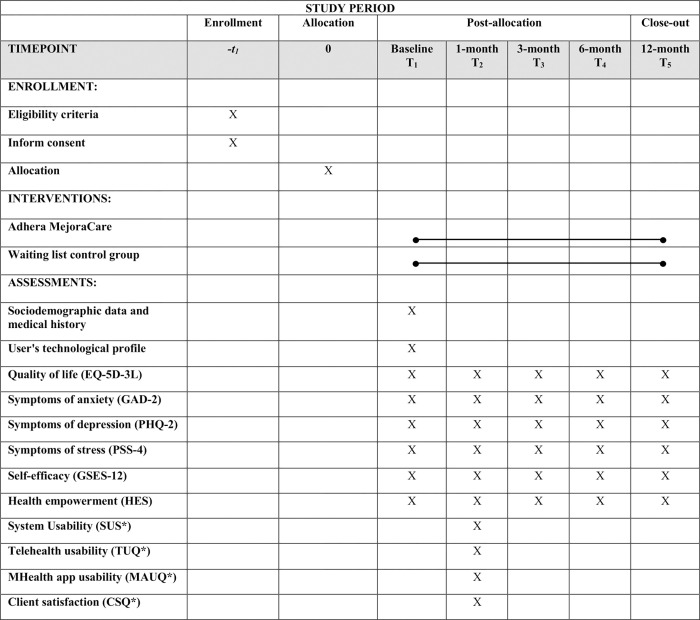
SPIRIT flow diagram: Schedule of enrollment, interventions and assessments. CFS, Computer Fluency Scale; EQ-5D-3L, EuroQol 5-Dimensions 3-Levels Questionnaire; GAD-2, Generalized Anxiety Disorder Questionnaire-2; PHQ-2, Patient Health Questionnaire-2; PSS-4, Perceived Stress Scale-4; GSES-12, General Self-Efficacy-12; HES, Health Empowerment Scale; SUS, System Usability Scale; TUQ, Telehealth Usability Questionnaire; MAUQ, mHealth App Usability Questionnaire; and CSQ, Client Satisfaction Questionnaire. *This instrument will be administered in the Adhera MejoraCare condition.

The general practitioners and specialists participating in the study will identify patients belonging to the high-risk group for CDs. Once identified, the physicians will make a face-to-face appointment with the potential candidates to explain the conditions of participation in the research study. Those interested in participating will sign the informed consent form and complete a brief questionnaire to verify compliance with the inclusion criteria: men and women; 18 years and older; CD diagnosis (i.e., cardiovascular disease, chronic obstructive pulmonary disease, diabetes, hypertension, and cancer); no severe psychiatric comorbidities (i.e., substance dependence, bipolar affective disorder, obsessive compulsive disorder, psychotic illness); and own an Android smartphone. Physicians participating in the study will have access to the patient’s medical history to check for the presence of a diagnosis of mental illness and the presence of severe psychiatric comorbidity. Subsequently, those who meet the criteria for participation in the study will complete the measures corresponding to the baseline through the *LimeSurvey* platform via a link that will be sent to the participant’s email address. Afterwards, participants will be randomized to one of the two conditions (intervention or control). An independent researcher from Valencia University will generate a permuted block randomization with fixed and concealed block length (20 participants per block), and with an allocation ratio of 1:1, using a computerized random number generator (Random Allocation Software 2.0 [30]). For methodological reasons, participants will not be blind to the condition. All patients will be assessed at 1-, 3-, 6- and 12-months from the time of randomization via the *LimeSurvey* platform. Fig 1 shows SPIRIT flow diagram of the study.

The intervention or follow-up will be discontinued if: a) the participant withdraws consent; b) the RCT is discontinued (e.g., due to poor patient recruitment, technological problems with the app that delivers the intervention); and c) the principal investigator or project administrator determines the need to discontinue the RCT (e.g., due to ethical and privacy issues, funding problems).

### Ethics and auditing

The study follows the guidelines of the Helsinki Declaration [31]. All the researchers will follow the guidelines for Good Clinical Practice [32], and the Ethical Principles and Code of Conduct for Psychologists of the American Psychological Association [33]. As noted, all the participants will be volunteers, and they will not receive any compensation for their participation. They will sign informed consent once the study and its conditions have been explained. Participants will be able to withdraw from the study at any time, without giving any reason and with no consequences. This study was approved by the Ethics Committee of Research in Humans of the Ethics Commission in Experimental Research of University of Valencia, (date: December 4, 2020; register number: 1472070) and the Ethics Committee of the Autonomous University of Encarnación. Besides, the study was registered in the United States National Institute of Health Registration System with Clinical Trials Registration Number: NCT04659746 (https://clinicaltrials.gov/ct2/show/NCT04659746). No audit has been planned at this time.

### Intervention

#### Experimental group

Adhera® MejoraCare Digital Program is an mHealth solution designed to raise awareness, educate and empower patients in the management of CD in the presence of the COVID-19, promoting behavioral changes in their lifestyle and emotional coping. A holistic "empowerment" approach based on cognitive-behavioral techniques (CBT) will be adopted to activate and empower CD management in patients. It will include evidence-based CBT techniques aimed at intervening on 1) cognitive, 2) emotional, and 3) behavioral variables, in three areas: COVID-19 psychoeducation, well-being, and lifestyle, following the guidelines from the World Health Organization for COVID-19 [34], Ryff’s well-being model [35] and health behaviour models for behaviour change [36]. Table 1 shows the contents included in the Adhera® MejoraCare Digital Program.

**Table 1:** Contents of the Adhera® MejoraCare Digital Program.

Module Topic	Subtopics
**COVID-19**	• What is the new coronavirus and COVID-19
	• Prevention measures for COVID-19
	• Day-to-day prevention
	• People at risk for COVID-19
	• If you have symptoms: quarantine
	• Care of the quarantined patient
	• Children and adolescents in confinement
**Well-being**	• Emotional management during confinement
	• Coping with grief
**Lifestyle**	• Healthy nutrition under challenging times
	• Physical activity at home

**Fig 2:**
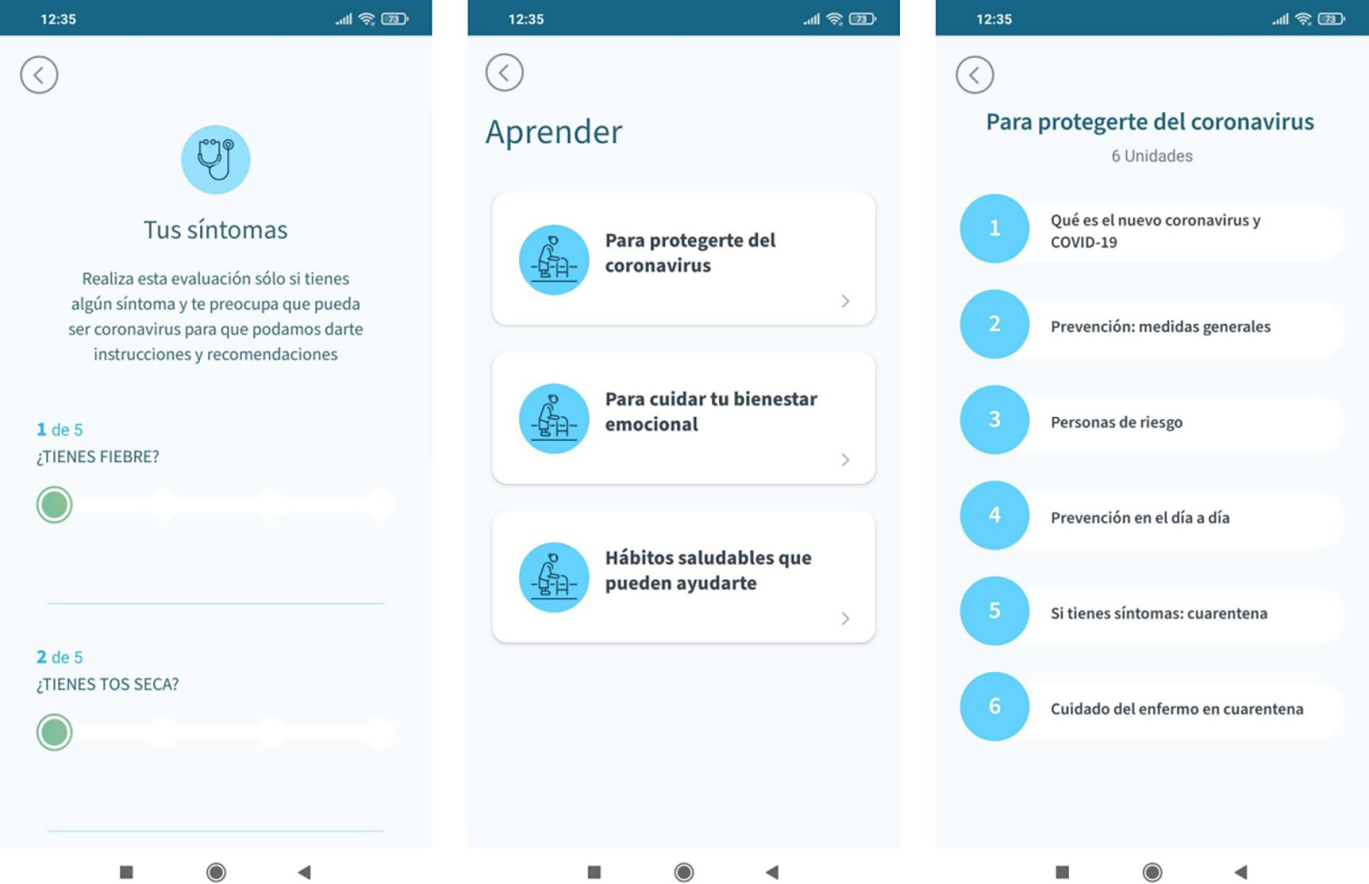
Functionalities of the Adhera MejoraCare solution. Left: Symptom’s monitoring. Center: Patient empowerment categories in the educational section. Right: Educational units included in the ‘‘How to protect from coronavirus” category.

Adhera® MejoraCare Digital Program includes the following functionalities (Fig 2): a) COVID-19 symptom monitoring; b) patient empowerment components (including educational methods and instructions to promote behavioral changes to establish and maintain a healthy lifestyle that minimizes the risk of contagion/propagation of the disease and improves patients’ quality of life); c) self-assessment tests to check and reinforce acquired knowledge; and d) tailored motivational messages that help to acquire lifestyle habits to prevent the spread of the COVID-19.

An AI-driven algorithm will be used for selecting and delivering tailored messages based on the user’s profile and scientific evidence, following the integrated behavior change model "I-Change". This algorithm is based on the work of Hors-Fraile et al. [37], and it has been successfully clinically validated for promoting smoking cessation in a randomized clinical trial [38].

The mobile app included in this mHealth solution will be downloaded and installed like any other commercial app from Google Play. The participant will receive an email from the research team with instructions on how to install the application. Specifically, the download link and an individual access code will be provided. The code can only be used once, which ensure that only one device can be use which the specific code. In case that the participant changed the mobile device during the study they can request an additional code, and this change will be track down to prevent data lose.

#### Control group

The WL group will not receive any intervention during the 1-year study period.

### Measures

#### Screening related measures

*Sociodemographic variables and medical history*. Personal data will be collected, including information such as age, gender, profession, marital status, employment status, level of studies, diagnosis of CD and its duration, clinical course, medication, diagnosis of mental disorder, and pregnancy.

*User’s technological profile*. The Computer Fluency Scale (CFS; [39]) is a self-report scale that assesses computer comfort and self-report of computing ability through 7 items rated on a 5-point Likert scale ranging from 1 (strongly disagree) to 5 (strongly agree). A high score indicates greater comfort with computers.

*Ad hoc* questionnaire designed to evaluate the frequency and the perceived ability to use a mobile phone. It is composed of 9 items (e.g., To what extent do you feel capable of using technologies? Have you ever used a computer?).

#### Primary outcome measure

*Quality of life*. The EuroQol 5-Dimensions 3-Levels Questionnaire (EQ-5D-3L; [40, 41]) is a self-report questionnaire that measures health-related quality of life and enables conversion to utility scores to calculate Quality-Adjusted Life-Years (QALYs) [42, 43]. It measures five dimensions of health: mobility, self-care, usual activities, pain/discomfort, and anxiety/depression. Each category consists of 1 item, ranging from 1 (no problems) to 3 (lots of problems). The second part of the EQ-5D-3L is a 20-centimetre vertical Visual Analogue Scale (VAS), ranging from 0 (worst imaginable health) to 100 (best imaginable health). The individual must mark the point on the scale that best reflects the assessment of their global health status today. The VAS’s use provides a complementary score to the descriptive system of the self-assessment of the individual’s health status [44].

#### Secondary outcome measures

*Anxiety symptoms*. The Generalized Anxiety Disorder Questionnaire-2 (GAD-2; [45, 46]) is a self-report measure that contains 2 items that assess anxiety symptoms. The items are scored on a 4-point Likert scale, ranging from 0 (no days) to 3 (almost every day), where a high score indicates greater anxiety symptoms.

*Depressive symptoms*. The Patient Health Questionnaire-2 (PHQ-2; [47, 48]) is a self-report measure that consists of 2 items that assess depressive symptoms. The items are scored on a 4-point Likert scale, ranging from 0 (never) to 3 (almost every day), where a high score indicates greater levels of depressive symptoms.

*Stress*. The Perceived Stress Scale-4 (PSS-4; [49, 50]) is a self-report scale that consists of 4 items that measures the degree to which life situations are considered stressful. The items are scored on a 4-point Likert scale, ranging from 0 (never) to 4 (very often), where a high score indicates a high perception of stress.

*Self-efficacy*. The General Self-Efficacy Scale-12 (GSES-12; [51, 52]) is a self-report questionnaire consisting of 12 items grouped into three factors: initiative (willingness to initiate behaviour), effort (willingness to make an effort to complete the behaviour) and persistence (persevering to complete the task in the face of adversity). The items are scored on a 5-point Likert scale, ranging from 1 (it never happens to me) to 5 (it always happens to me), where a high score indicates higher levels of self-efficacy.

*Health empowerment*. The Health Empowerment Scale (HES; [53]) is a self-report measure that assesses health-related empowerment (e.g., health-related satisfaction, goal achievement, stress management, social support, self-motivation, decision making). It consists of 8 items scored on a 5-point Likert scale ranging from 1 (strongly disagree) to 5 (strongly agree), where high scores are indicators of a higher level of health-related empowerment.

#### mHealth solution usability and treatment satisfaction

*System usability*. The System Usability Scale (SUS; [54, 55]) is a self-reported measure that assesses the intervention’s overall usability. It is a 10-item scale, measured on a 5-point Likert scale ranging from 1 (strongly disagree) to 5 (strongly agree). Total SUS scores range from 0 to 100. The questionnaire is designed to be answered after the user’s interaction with the system.

*Telehealth usability*. An adaptation of the original Telehealth Usability Questionnaire (TUQ; [56, 57]) will be used in this study. This self-report questionnaire provides information on the telehealth system’s usability (usefulness, ease of use, effectiveness, reliability and satisfaction). It consists of 16 items on a 7-point Likert scale that range from 1 (strongly disagree) to 7 (strongly agree). The total score ranges from 7 to 112. Higher scores are indicators of higher usability of the telehealth system.

*mHealth solution usability*. The mHealth App Usability Questionnaire (MAUQ; [58]) is a self-reported instrument that assesses the usability of mHealth solutions. It consists of 26 items with 3 subscales (ease of use, interface and satisfaction, usefulness) on a 7-point Likert scale ranging from 1 (strongly disagree) to 7 (strongly agree). The total score ranges from 21 to 147. High scores are indicators of higher usability of mHealth solutions.

*Client satisfaction*. The Client Satisfaction Questionnaire (CSQ; [59–61]) is a self-reported questionnaire that assesses the general satisfaction of the patient with health and human services. It consists of 8 items on a 4-point Likert scale ranging from 1 (lowest score per item) to 4 (highest score per item). The total score ranges from 8 to 32, with higher values indicating higher satisfaction.

#### Adhera® MejoraCare Digital Program adherence

The number of times the participant uses the mHealth solution will be recorded and tracked.

### Data management

Online data collected from LimeSurvey will be stored at protected servers of the University of Valencia. Only individuals authorized by the principal investigator will have access to the database. After completing the assessment points, authorized individuals will download the database in a SPSS format that will be automatically saved onto a password-protected cloud server of the University of Valencia (nÚVol, https://nuvol.uv.es). Pseudonymization will be used for the processing of participants’ personal data while the study is active. Once the study has finished, an anonymized copy of the database will be stored at the University of Valencia for up to 5 years after the project ends, following the principles of the European GDPR 2016/679.

### Analysis

First, descriptive statistics and normality assumptions will be examined. In case assumptions are met, t-tests for independent samples, ANOVAs, and chi-square analysis will be calculated to observe differences between groups at baseline in quantitative and categorical variables. In case normality assumptions are not met, robust t-test for independent samples and robust ANOVAs will be calculated [62]. Effect size will be quantified using Cohen’s d (d; [63]) or its robust equivalent (*d*_*R*_; [64]) when normality assumptions are not met, and robust estimations are employed. Independent-sample t-tests and chi-squared tests will be also performed to check that there are no significant differences in sociodemographic characteristics of the participants between groups that can act as confounding variables (i.e., age, gender, year and CD diagnosis).

Then, the multivariate normality assumption on the primary and secondary outcome measures will be tested before computing the linear mixed models. If the assumption is met, linear mixed models will be performed for each dependent variable, considering temporal moments as an intra-group factor and condition as an inter-group factor, using the False Discovery Rate (FDR) adjustments for post-hoc multiple comparisons. Otherwise, their mixed robust equivalents will be calculated [62].

In addition, multiple hierarchical regression models will be used to identify possible predictors of evolution in the primary and secondary measures. In particular, it is expected that characteristics as the gender, age, the CD, and user’s technological profile will act as predictor for the level of adherence to Adhera® MejoraCare Digital Program intervention, and the change in quality of life, self-efficacy, health empowerment, and anxiety and depression symptomatology. Finally, moderation analyses will be performed to test whether the gender, age, the CD, user’s technological profile and level of adherence moderate the intervention’s effect on quality of life, self-efficacy, health empowerment, and anxiety and depression symptomatology. These analyses will be performed using the macro PROCESS (version 4.0, https://processmacro.org/). The conditional effects of condition on the dependent variables at medium (the mean), low (−1 SD), and high (+1 SD) levels of the moderator variables will be examined with the “pick-a-point approach”.

### Data monitoring

The study will not have a formal data monitoring committee. Any unexpected adverse events or outcomes will be discussed by the trial management committee (identical to the authors of this protocol). In addition, the trial management committee will monitor recruitment, treatment and attrition rates, as well as any study-related concerns.

### Access to data

Prior to publication of the main results, the principal investigators will have access to the complete data set. The principal investigators will address data issues and finalize the data set for statistical analysis. After publication, only the principal investigator and persons approved by the principal investigator will have access to the data set.

### Dissemination and protocol amendments

The RCT results will be submitted for publication to an international, peer-reviewed journal, regardless of whether the results are positive, negative or inconclusive in relation to the study hypothesis.

Any important protocol amendments will be reported to the Ethics Committee of Research in Humans of the Ethics Commission in Experimental Research of University of Valencia, registered at ClinicalTrials.gov and communicated in the primary RCT report.

## Discussion and conclusion

In the present paper, we described the protocol for a RCT to test the efficacy of the Adhera® MejoraCare Digital Program intervention, an mHealth solution designed to provide information about COVID-19 and promote well-being and a healthy lifestyle in CD patients from Paraguay. Mainly, it is expected that the digital intervention group will be more effective than the WL group in increasing the quality of life and decreasing the emotional distress (anxiety, depression, and stress) of CD patients during the COVID-19 outbreak and that these changes will be maintained in the follow-ups. It is also expected that the Adhera® MejoraCare Digital Program will be well rated in terms of usability and satisfaction.

This study stands out for an integrative and holistic approach to addresses the care of chronic conditions, including access to treatment, monitoring of health behaviors, and emotional support through the use of digital interventions, especially in times of social distancing and movement restriction measures during the COVID-19 period [65].

To our knowledge, this is the first RCT in Paraguay, which uses an mHealth solution intervention in a clinical population for the management of CD. In this regard, a study’s strength is the response to the emergence of COVID-19 in a low- middle-income country (LMIC). The global crisis in well-resourced countries (e.g., United States, United Kingdom) in containing this pandemic raises concerns about the impact of COVID-19 in LMICs. In this regard, this work’s results will provide relevant evidence on the use of technological solutions to complement primary care services and reduce inequalities in access to long-term care [66]. So, it is expected that the results will contribute with evidence to transform clinical practice and access to healthcare in countries with fragile health systems.

We would like to mention some possible limitations. First, the possible presence of a high dropout or attrition rate by participants from the mHealth solution intervention condition [67]. Second, the results of this RCT should be interpreted with caution as they are limited to only a few specific groups of CDs. Future research with mHealth solutions should focus on increasing the sample size and performing in-depth analyses on a wide range of CDs to draw more robust and generalized conclusions. Third, implementing mHealth technologies in a LMIC will represent a major challenge for both researchers and users given the characteristics of the context (e.g., Internet connectivity issues, experience with technology). Finally, it is worth mentioning that this study only includes participants with an Android smartphone, thus limiting the recruitment of potential users with smartphones with different operating systems such as iOS.

In conclusion, this study’s results could help understand better how to offer self-care strategies to people with CD and shed evidence on the impact of mHealth solutions on the management of chronic medical conditions in a LMIC during the COVID-19 pandemic.

## Supporting information

S1 File SPIRIT checklist. (PDF)

S2 File Ethics committee protocol. (PDF)

S3 File (DOCX)
