# Correction: mHealth intervention to improve quality of life in patients with chronic diseases during the COVID-19 crisis in Paraguay: A study protocol for a randomized controlled trial

**DOI:** 10.1371/journal.pone.0303726

**Published:** 2024-05-09

**Authors:** The PLOS One Staff

The affiliation for the 12^th^ author is incorrect. Marta Miragall is not affiliated with #4 but with #5: Department of Personality, Evaluation and Psychological Treatment, Faculty of Psychology, University of Valencia, Valencia, Spain.

The publisher apologizes for the error.

## References

[1] Escrivá-Martínez T, Vara MD, Czeraniuk N, Denis M, Núñez-Benjumea FJ, Fernández-Luque L, et al. (2022) mHealth intervention to improve quality of life in patients with chronic diseases during the COVID-19 crisis in Paraguay: A study protocol for a randomized controlled trial. PLoS ONE 17(11): e0273290. doi: 10.1371/journal.pone.0273290 36346807 PMC9642890

