# Correction: mHealth intervention to improve quality of life in patients with chronic diseases during the COVID-19 crisis in Paraguay: A study protocol for a randomized controlled trial

**DOI:** 10.1371/journal.pone.0345669

**Published:** 2026-03-24

**Authors:** Tamara Escrivá-Martínez, Mª Dolores Vara, Nadia Czeraniuk, Matías Denis, Francisco J. Núñez-Benjumea, Luis Fernández-Luque, Alba Jiménez-Díaz, Vicente Traver, Juan-José Lull, Antonio Martínez-Millana, Jorge Garcés-Ferrer, Marta Miragall, Rocío Herrero, Analía Enríquez, Verena Schaefer, Sergio Cervera-Torres, Cecilia Villasanti, Carmen V. Cabral, Irene Fernández, Rosa Mª Baños

The ninth author’s name is spelled incorrectly. The correct name is: Juan-José Lull.
