# Supplementary material for: mHealth intervention to improve quality of life in patients with chronic diseases during the COVID-19 crisis in Paraguay: A study protocol for a randomized controlled trial
Source: PLoS One. 2022 Nov 8;17(11):e0273290. doi: 10.1371/journal.pone.0273290 (PMC9642890; doi:10.1371/journal.pone.0273290)
Supplement: S2 File — (PDF) [file pone.0273290.s002.pdf]

FORMULARIO EH-1
SOLICITUD DE INFORME de APROBACION AL COMITÉ DE ÉTICA de INVESTIGACIÓN en HUMANOS

**DATOS DEL PROYECTO DE INVESTIGACIÓN**
**TÍTULO:** Tecnología digital para optimizar la salud mental y el bienestar en pacientes crónicos durante la crisis de la COVID-19 en Paraguay.
**DURACIÓN DEL PROYECTO:** 12 meses
**ESPECIFICAR SI EL PROYECTO HA SIDO O NO EVALUADO POR OTRO COMITÉ:** No
**CARACTERÍSTICAS DEL PROYECTO**
**JUSTIFICACIÓN DEL PROYECTO:**<br>La pandemia del coronavirus, causada por el virus SARS-CoV-2, ha desencadenado una alerta sanitaria a nivel mundial y ha ido propagándose rápidamente por el mundo desde su aparición. Según la OMS, la mortalidad producida por enfermedades infecciosas, como la COVID-19, se produce con mayor frecuencia en poblaciones vulnerables de riesgo, entre las que destacan personas de edad avanzada o pacientes con enfermedades crónicas previas (EPOC, cáncer, diabetes, enfermedades cardíacas, etc.). En Paraguay, según el portal oficial del Ministerio de Salud Pública y Bienestar Social, el día 15 de abril se registraron 174 casos, con 8 fallecidos y 30 recuperados, tras lo cual el gobierno implementó medidas de prevención, intervención y

estrategias de mitigación (suspensión de eventos masivos, horario excepcional de trabajo, restricción de movilidad nocturna, cierre de fronteras, etc.), lo que ha llevado a Paraguay a una situación de confinamiento estricto.

La pandemia de la COVID-19 y las medidas tomadas para controlar su propagación (confinamiento, cese de actividades, limitación de movimientos) están teniendo un enorme impacto a nivel sanitario, social y económico, pero también psicológico. Los estudios publicados recientemente (Brooks et al., 2020; Duan & Duan, 2020; Montemuro, 2020) señalan que estas medidas, junto con la situación que genera la pandemia (riesgo de enfermar, posibilidad de perder el trabajo,...) tendrán un impacto negativo en la salud mental, incrementándose las tasas de estrés y ansiedad, así como de otros problemas psicológicos (irritabilidad, depresión,...) en la población en general, y más especialmente, en las personas vulnerables y/o de riesgo (mayores de 60 años, enfermos crónicos, sanitarios,...). Además, se espera que estos problemas se exacerben, y si estos efectos negativos no se tratan de manera precoz, podrían prolongarse incluso finalizada la cuarentena y la pandemia. Por tanto, es necesario contar con herramientas psicológicas eficaces para combatir los problemas psicológicos y emocionales generados por el brote de la COVID-19, dirigidos a grupos especialmente vulnerables, como los pacientes con enfermedades crónicas.

Los estudios realizados hasta el momento sobre los efectos de la pandemia y el confinamiento sobre la salud mental señalan que esta situación incrementa de manera significativa los niveles de estrés, produciendo un aumento en ansiedad, depresión, soledad, etc., con consecuencias negativas que pueden llevar al desarrollo de trastornos mentales (Brooks et al., 2020; Duan & Duan, 2020; Montemuro, 2020). Además, las constantes noticias sobre el virus, junto con las medidas restrictivas que se han de tomar para reducir su tasa de contagio, suponen un gran estrés emocional para toda la población, pero especialmente para los grupos de riesgo, como las personas con enfermedad crónica, en los cuales incluso puede agravarse su condición crónica, derivando en una peor gestión de su enfermedad crónica y colapsar los servicios sanitarios innecesariamente si acuden ante cualquier aparente síntoma. Por ello, se necesita un refuerzo y guía conductual que permita a las personas con enfermedades crónicas conocer y entender las medidas necesarias para prevenir el contagio, o actuar si se está contagiado, a la vez que es necesario ofrecer a estas personas herramientas que las ayuden a prevenir problemas psicológicos y emocionales que puedan empeorar su situación médica y su calidad de vida.

Los estudios con relación a la instauración y mantenimiento de nuevas conductas de salud demuestran que no alcanza con aportar la información necesaria al paciente. Esta información es un componente necesario y fundamental, pero no suficiente para que se produzca este cambio de conducta. Las teorías desarrolladas para estudiar el comportamiento humano postulan la existencia de diferentes fases y factores (sociodemográficas, culturales, sociales…) para que se produzca un cambio efectivo en el comportamiento. Dentro de los modelos, el modelo de Cambio de Comportamiento Integrado (I-Change) (De Vries, 2017) integra las contribuciones de modelos como: a) el modelo de Actitud - Influencia social – Autoeficacia; b) la Teoría del Comportamiento Planificado; c) la Teoría Cognitiva Social; d) el establecimiento de metas; e) el modelo de Salud-Creencia; y f) el modelo de Cambio de Comportamiento Transteórico (De Vries, 2018; De Vries, et al., 2018; De Vries, Mudde, & Dikstra, 2000). El modelo I-Change propone que el cambio de comportamiento tiene cuatro pasos: *concienciación, motivación, acción, y el comportamiento adquirido*. Las personas pasan del primero al cuarto en base a diferentes determinantes de conducta en cada fase, que están condicionados por factores de externos de información (canal, fuente, mensaje e interlocutor)

que pueden ser modificados, y factores internos (biológicos, psicológicos, conductuales, ambientales) que no pueden ser modificados.

Por ello, educar y empoderar conductualmente y emocionalmente a los pacientes crónicos en situaciones de riesgo de contagio es fundamental, ya que disponer de información relevante y de estrategias adecuadas y eficaces, pueden facilitar que los pacientes se involucren en acciones específicas (conductas de salud) y en cambios comportamentales, actitudinales y emocionales (p.ej., medidas de higiene personal, seguimiento de prescripciones terapéuticas, etc.) que redunden en un mejor manejo de la salud y en su calidad de vida. Además, la enseñanza y entrenamiento en estrategias e intervenciones psicológicas eficaces pueden ayudar a estos pacientes a manejar de una manera más eficiente su malestar emocional y mejorar su calidad de vida.

Tras el brote de la COVID-19, las herramientas digitales se han vuelto fundamentales para detectar, contener y buscar tratamientos y apoyos para la enfermedad. Estas herramientas presentan una serie de ventajas con respecto a los tratamientos presenciales tradicionales, como: a) accesibilidad; b) uso flexible en cualquier momento y lugar; c) adaptabilidad al ritmo del usuario; d) anonimato; e) contenido estandarizado, que permite avanzar sin la presencia del terapeuta; f) contenido adaptado a la población objetivo; y f) bajos costos en la prestación de servicios de salud mental (Schröder et al., 2016). Como señala el reciente artículo publicado en la prestigiosa revista JMIR (Torous et al, 2020), la pandemia ha hecho aún más evidente el potencial de la salud digital para aumentar el acceso y la calidad de los servicios de salud mental, y se puede afirmar que en estos momentos las soluciones de “telesalud” son la solución correcta y adecuada para brindar atención en salud mental en la crisis actual. Las aplicaciones de salud mental representan una oportunidad para ampliar el acceso y la calidad de los tratamientos psicológicos (Anthes, 2016). Las distintas organizaciones e instituciones relacionadas con la atención en salud mental señalan que este tipo de tecnología puede proporcionar soluciones rentables y escalables a la brecha actual del tratamiento (East y Havard, 2015). En concreto, las aplicaciones móviles pueden ser una herramienta útil para ofrecer tratamientos psicológicos, en comparación con otras plataformas, debido a: a) facilidad de hábito, b) baja expectativa de esfuerzo, y c) alta motivación hedónica (East & Havard, 2015; Yuan et al., 2015). Además, las aplicaciones de salud mental pueden abarcar todas las etapas de la prestación de atención clínica, desde la prevención hasta el manejo de la condición después del tratamiento (es decir, la prevención de recaídas) (Price et al., 2014).

Por tanto, las soluciones mediante tecnologías móviles hechas mediante un canal de actuación seguro, con un alto nivel de penetración en la población, escalables, y con un coste relativo bajo, las hacen perfectas como herramienta para realizar estrategias de salud pública en este ámbito. Además, la falta de soluciones digitales específicas para educar y empoderar a pacientes crónicos en la prevención de enfermedades infecciosas como la COVID-19 y otras enfermedades pandémicas obligan a que estos pacientes tengan que acudir a los centros de salud y hospitales con frecuencia, aumentando el riesgo de infección. Al tratarse de enfermedades de rápida transmisión, la prevención juega un papel importante y la transmisión de los virus presenta riesgos especiales dentro de las instalaciones de atención médica y puede causar brotes explosivos de enfermedades ocasionando graves problemas al sistema sanitario.

Este proyecto de investigación propone el uso de las tecnologías para dispensar un programa de apoyo psicológico dirigido específicamente a una población particularmente vulnerable durante la pandemia: los enfermos crónicos. El programa, denominado “Mejora.CareTM”, ha

sido desarrollado por Saludmedia Labs en España y forma parte de la plataforma AdheraHealthTM. Dicha plataforma ha mostrado su eficacia como herramienta para ofrecer apoyo emocional y promover cambios conductuales en distintos contextos clínicos (Carrasco et al., 2020; Luna-Perejon et al., 2019; Hors-Fraile et al., 2019).

Mejora.CareTM está dirigido a ofrecer apoyo a pacientes con enfermedades crónicas. Por un lado, se brindará información educativa que les ayude a comprender mejor su enfermedad, a seguir las indicaciones médicas y a mejorar sus conductas de prevención del contagio. Y, por otro lado, se proporcionarán herramientas para el autocuidado y la gestión emocional del estrés, la ansiedad y otras emociones negativas generadas por la crisis pandémica y sus consecuencias, especialmente en la población diana de este estudio, ya que es considerada una población de alto riesgo. En este sentido, la tecnología propuesta permitirá concienciar, educar y empoderar a los pacientes crónicos, fomentando cambios de conducta que promuevan el autocuidado para reducir los riesgos de contagio de la COVID-19. Al mismo tiempo, ofrecerá herramientas de manejo emocional con el objetivo de mejorar su salud mental, calidad de vida y bienestar emocional.

Mejora.CareTM ofrece a los pacientes los siguientes componentes:
a) monitorización de síntomas; b) componentes de empoderamiento del paciente (incluyendo métodos educativos, ejercicios de entrenamiento para la gestión de ansiedad y emociones negativas, e instrucciones para promover cambios de conducta que instauren y/o mantengan un estilo de vida saludable que minimice el riesgo de contagio/propagación de la enfermedad y mejore la calidad de vida de los pacientes); c) acceso a información relacionada emitida desde fuentes oficiales; y d) acceso a mapa de recursos sanitarios.

El programa también ofrece herramientas a los proveedores de salud, como son:
-La segmentación y seguimiento de potenciales pacientes de COVID-19 y personas de alto riesgo: incluyendo herramientas de seguimiento a través de mensajes que pueden personalizarse según las necesidades de un paciente en concreto o de un grupo determinado.
- Apoyo a pacientes en fase de recuperación domiciliaria: incluyendo herramientas para la monitorización en tiempo real de síntomas, seguimiento del empeoramiento o mejoría, comunicación a través de mensajes para brindar apoyo, consejos o instrucciones personalizadas.

En resumen, esta herramienta tecnológica permitirá recoger datos reales de nivel de conocimiento, conductas, y síntomas, que permitirán analizar y mejorar la toma de decisiones de las autoridades sanitarias. Se espera que la utilización de esta tecnología digital tenga un gran impacto en la calidad de vida de pacientes con enfermedades crónicas, así como también una mejora en la optimización de los servicios ofrecidos por el sistema de salud pública. También se espera que la plataforma contribuya a concienciar y educar a los pacientes, permitiendo asistirles y ofrecerles apoyo emocional sin la necesidad de asistir a los centros de salud y hospitales.

Referencias bibliográficas

Andersson, G. & Titov, N. (2014). Advantages and limitations of Internet-based interventions for common mental disorders. *World Psychiatry, 13*(1), pp.4-11.
Andrews, G., Basu, A., Cuijpers, P., Craske, M.G., McEvoy, P., English, C.L., & Newby JM. (2018). Computer therapy for the anxiety and depression disorders is effective,

acceptable and practical health care: an updated meta-analysis. *Journal of Anxiety Disorders, 55*, 70-78.
Anthes, E. (2016). Mental health: There's an app for that. *Nature, 532*(7597), 20-23.
Botella, C., Quero, S., Baños, R. & García-Palacios, A. (2009). Avances en los tratamientos psicológicos: la utilización de las nuevas tecnologías de la información y la comunicación. *Anuario de psicología/The UB Journal of Psychology, 40*(2), pp.155-170.
Brooks, S.K., Webster, R.K., Smith, L.E., Woodland, L., Wessely, S., Greenberg, N., & Rubin, G.J. (2020). The psychological impact of quarantine and how to reduce it: rapid review of the evidence. *The Lancet, 395*(10227). https://doi.org/10.1016/S0140-6736(20)30460-8
Carrasco-Hernández, L., Jódar-Sánchez, F., Nuñez-Benjumea, F., Moreno-Conde, J., Mesa-González, M., Civit-Balcells, A., Hors-Fraile, S., … Ortega-Ruiz, F.A. (2020) Digital Therapeutics Solution Complementing Psychopharmacology-Supported Smoking Cessation: Randomized Controlled Trial. *JMIR mHealth*. DOI: 10.2196/17530
De Vries, H. (1998). Planning and evaluating health promotion. Evaluating health promotion.
De Vries, H. (2017). An integrated approach for understanding health behavior: The I-Change Model as an example. *Psychology and Behavioral Science International Journal,* 2(2).
De Vries, H., et al., (1998). Differential beliefs, perceived social influences, and self-efficacy expectations among smokers in various motivational phases. *Preventive Medicine*, *27*(5): p. 681-689.
De Vries, H., Mudde, A.& A. Dijkstra (2000). The attitude-social influence-efficacy model applied to the prediction of motivational transitions in the process of smoking cessation. Understanding and changing health behaviour: From health beliefs to self-regulation, p. 165-187.
Duan, L., & Zhu, G. (2020) Psychological interventions for people affected by the COVID-19 epidemic. *Lancet Psychiatry. 7*(4), 300-302.
East, M.L., & Havard, B.C. (2015). Mental health mobile apps: from infusion to diffusion in the mental health social system. *JMIR Mental Health, 2(*1), e10.
Giwa, A.L., Desai, A., & Duca, A. (2020) Novel 2019 Coronavirus SARS-CoV-2 (COVID-19): An Updated Overview for Emergency Clinicians. *Emerg Med Pract; 21(*5): 1-28.
Hors-Fraile. S., Vries, H., Malwade, S., Luna-Perejon, F., Amaya, C., Civit, A., ... & Li, Y. C. (2019) Opening the Black Box: Explaining the Process of Basing a Health Recommender System on the I-Change Behavioral Change Model, in IEEE Access, vol. 7, pp. 176525-176540. doi: 10.1109/ACCESS.2019.2957696
Kazdin, A.E. & Blase, S.L. (2011). Rebooting psychotherapy research and practice to reduce the burden of mental illness. *Perspectives on Psychological Science, 6*(1), pp.21-37.
Kazdin, A.E. & Rabbitt, S.M. (2013). Novel models for delivering mental health services and reducing the burdens of mental illness. Clinical Psychological Science, 1(2), pp. 170-191.
Luna-Perejon, F., Malwade, S., Styliadis, C., Civit, J., Cascado-Caballero, D., Konstantinidis, E., ...Li, Y.J. (2019) Evaluation of user satisfaction and usability of a mobile app for smoking cessation. *Comput Methods Programs Biomed, 182*:105042. doi: 10.1016/j.cmpb.2019.105042
Ministerio de Salud Pública y Bienestar Social, Dirección General de Vigilancia de la Salud. Boletín de Vigilancia N3 Enfermedades No Transmisibles y Factores de Riesgo, 2019. Asunción.
Ministerio de Salud Pública y Bienestar Social, Dirección General de Vigilancia de la Salud, 2017. Política Nacional de Calidad en Salud 2017-2030. ISBN 978-99967-36-61-2
Montemurro N. (2020) The emotional impact of COVID-19: From medical staff to common people. *Brain Behav Immun*, S0889-1591(20) 30411-6. doi:10.1016/j.bbi.2020.03.032
Organización Mundial de la Salud. (2020) Statement on the second meeting of the International Health Regulations (2005) Emergency Committee regarding the outbreak of novel

coronavirus (2019-nCoV). Nota de prensa. Disponible en: https://www.who.int/news-room/detail/30-01-2020-statement-on-the-second-meeting-of-the-international-health-regulations-(-005)-emergency-committee-regarding-the-outbreak-of-novel-coronavirus-(2019-ncov)

Price, M., Yuen, E. K., Goetter, E. M., Herbert, J.D., Forman, E.M., Acierno, R., & Ruggiero K.J. (2014). mHealth: a mechanism to deliver more accessible, more effective mental health care. *Clinical Psychology & Psychotherapy, 21*(5),427-436.

Schröder, J., Berger, T., Westermann, S., Klein, J. P., & Moritz, S. (2016). Internet interventions for depression: new developments. *Dialogues in Clinical Neuroscience, 18*(2), 203-212.

Torous, J., Jän Myrick, K., Rauseo-Ricupero, N., & Firth, J. (2020) Digital Mental Health and COVID-19: Using Technology Today to Accelerate the Curve on Access and Quality Tomorrow. *JMIR Ment Health, 7*(3):e18848. doi:10.2196/18848

Yuan, S., Ma, W., Kanthawala, S. & Peng, W. (2015). Keep using my health apps: discover users'perception of health and fitness apps with the UTAUT2 model. *Telemedicine and e-Health, 21*(9), 735-741.

OBJETIVOS DE LA INVESTIGACIÓN:

El **objetivo principa**l de este proyecto es estudiar los efectos de una intervención (Mejora.CareTM) dirigido a ofrecer apoyo e información, y entrenar habilidades de empoderamiento en pacientes con enfermedades crónicas (EPOC, cáncer, diabetes, enfermedades cardíacas. hipertensión, etc.) durante la crisis de la COVID-19.

Este objetivo se desglosa en los siguientes **objetivos específicos:**

1. Evaluar la eficacia del uso de la herramienta Mejora.CareTM en la mejora de los niveles de calidad de vida y bienestar emocional y la reducción de sintomatología de ansiedad y depresión en pacientes con enfermedades crónicas en comparación con un grupo control.
2. Evaluar la aceptación de la herramienta Mejora.CareTM por parte de los usuarios.

Las **hipótesis de investigación** son las siguientes:

Hipótesis 1. Se espera que los pacientes que utilicen la aplicación presenten un aumento en los niveles de calidad de vida y bienestar emocional en comparación con el grupo control en los seguimientos a 1, 3, 6 y 12 meses.

Hipótesis 2. Se espera que los pacientes que utilicen la aplicación presenten una reducción significativa en los niveles de ansiedad y depresión en comparación con el grupo control en los seguimientos a 1, 3, 6 y 12 meses.

Hipótesis 3. Los pacientes valorarán positivamente la utilidad de la aplicación y mostrarán niveles elevados de satisfacción y aceptación.

DISEÑO METODOLÓGICO: El diseño de este proyecto es un estudio controlado de dos brazos (grupo intervención y grupo control). El estudio se realizará con pacientes crónicos pertenecientes a los hospitales de la región de Itapúa y será realizado siguiendo las guías CONSORT (Consolidated Standards of Reporting Trials, http://www.consort-statement.org) (Moher et al., 2010), CONSORT-EHEALTH (Eysenbach, 2011) y las guías SPIRIT.

LUGAR DONDE SE DESARROLLARÁ LA INVESTIGACIÓN: Hospitales de la región de Itapúa de Paraguay.

TAMAÑO MUESTRAL:
En cuanto al tamaño de la muestra requerida, usando el programa G-Power (Faul et al., 2007), se ha establecido que son necesarios 39 participantes por condición (78 en total), teniendo en cuenta un tamaño del efecto esperado moderado en la medida principal de cambio (f = 0.25) según Liu et al. (2011) y Ramachandran et al. (2007), una potencia estadística de .80, y un alfa de .05. Previendo un posible abandono del 20% el tamaño mínimo total de la muestra se elevará a 94 pacientes.

CRITERIOS DE SELECCIÓN DE LOS PARTICIPANTES:
Los criterios de inclusión serán: a) ser mayor de 18 años; b) capacidad para entender y leer español; c) disponer de un teléfono móvil tipo smartphone con conexión a Internet; d) estar diagnosticado de una enfermedad crónica (i.e., EPOC, cáncer, diabetes, enfermedades cardíacas, hipertensión); e) proporcionar consentimiento informado por escrito.

Los criterios de exclusión serán: a) carencias de conocimientos tecnológicos e incapacidad para utilizar el móvil; b) diagnóstico de trastornos psiquiátricos o deficiencia cognitiva severa.

VARIABLES RECOGIDAS EN LA INVESTIGACIÓN Y DESCRIPCIÓN DE LAS INTERVENCIONES A REALIZAR:

Medidas:

Variables sociodemográficas e historial médico
Se recogerán datos personales, incluyendo información como la edad, el sexo, la profesión, el estado civil, la situación laboral, el nivel de estudios, el diagnóstico de enfermedad crónica y su duración, el curso clínico, la medicación, el diagnóstico de trastorno mental y el embarazo (Línea base).

Perfil tecnológico del usuario
- La “Escala de Fluidez Informática” (CFS; Becker, 2012) (Línea de base).
- Cuestionario ad hoc diseñado para evaluar la frecuencia y la capacidad percibida para utilizar un teléfono móvil (Línea de base).

Medida de resultado primario
- **Calidad de vida:** Cuestionario “EuroQol” de 5 dimensiones y 3 niveles (EQ-5D-3L; Badia et al., 1999; Grupo EuroQol, 1999) (Línea base, 1, y 3, 6 y 12 meses).

Medidas de resultado secundarias
- **Síntomas de ansiedad**: El “Generalised Anxiety Disorder Questionnaire-2” (GAD-2; Kroenke et al., 2007; García-Campayo et al., 2012) (Línea base, 1, y 3, 6 y 12 meses).
- **Síntomas depresivos**: El “Patient Health Questionnaire-2” (PHQ-2; Kroenke et al., 2003; Rodríguez-Muñoz et al., 2017) (Línea base, 1, y 3, 6 y 12 meses).
- **Estrés:** La “Escala de Estrés Percibido-4” (PSS-4; Herrero et al., 2006; Vallejo et al., 2018) (Línea base, 1, y 3, 6 y 12 meses).
- **Autoeficacia:** La “Escala de Autoeficacia General-12” (GSES-12; Bosscher et al., 1997; Herrero et al., 2014) (Línea base, 1, y 3, 6 y 12 meses).

- Empoderamiento en salud: La “Escala de Empoderamiento en Salud” (HES; Serrani et al., 2014) (Línea base, 1, y 3, 6 y 12 meses).
- Usabilidad del sistema: La “Escala de Usabilidad del Sistema” (SUS; Brooke, 1996; Sevilla-González et al., 2020) (1 mes).”
- Usabilidad de la telesalud: Una adaptación del Cuestionario de Usabilidad de Telesalud original” (TUQ; Parmanto et al., 2016; Torre et al., 2020) (1 mes).
- Usabilidad de la aplicación: El “Cuestionario de usabilidad de aplicaciones de mSalud” (MAUQ; Attkisson et al., 1996) (1 mes).
- Satisfacción del cliente: The “Client Satisfaction Questionnaire” (CSQ; Larsen et al., 1979; Roberts et al., 1984) (1 mes).

Intervenciones:

Mejora.CareTM

Los participantes asignados al grupo intervención accederán a la aplicación Mejora.CareTM, a través de su dispositivo móvil o Smartphone. Esta aplicación móvil incluye las siguientes funcionalidades: a) monitorización de síntomas; b) componentes de empoderamiento del paciente (incluyendo métodos educativos, ejercicios de entrenamiento para la gestión de ansiedad y emociones negativas, e instrucciones para promover cambios de conducta que instauren y/o mantengan un estilo de vida saludable que minimice el riesgo de contagio/propagación de la enfermedad y mejore la calidad de vida de los pacientes); c) acceso a información relacionada emitida desde fuentes oficiales; y d) acceso a mapa de recursos sanitarios. La aplicación dispondrá de los siguientes contenidos:

- Contenidos educacionales de fácil comprensión sobre el nuevo coronavirus y cómo prevenir el contagio, así como información útil para el manejo de las emociones ante esta situación. Como parte del programa de aprendizaje, los contenidos incluyen tests de autoevaluación que permiten comprobar y afianzar los conocimientos adquiridos.
- Mensajes automatizados personalizados que ayudan a las personas a adquirir hábitos de vida para prevenir el contagio por el coronavirus. Los contenidos de los mensajes han sido validados por profesionales de la salud, expertos en cambio de comportamiento y comunicación y promoción de la salud.

Grupo control: Lista de espera (LE)

El grupo LE no recibirá ninguna intervención durante el periodo de estudio de 1 año.

Análisis de datos:
Se calcularán estadísticos descriptivos, pruebas t para muestras independientes, ANOVAs y análisis de chi-cuadrado para observar las diferencias entre los grupos en la línea de base en las variables cuantitativas y categóricas. Se realizarán modelos lineales mixtos para cada variable dependiente, considerando los momentos temporales como factor intragrupo y la condición como factor intergrupo. Se utilizará la corrección de Bonferroni para las comparaciones múltiples posthoc, y se calculará el tamaño del efecto d de Cohen para la comparación intra e intergrupal. Se utilizarán modelos de regresión jerárquica múltiple para identificar posibles predictores de la evolución en las medidas primarias y secundarias. Por último, se realizarán análisis de mediación y moderación para analizar las variables responsables del efecto de la intervención sobre las variables dependientes y detectar el perfil de los pacientes que más se beneficiarán de la intervención.

IMPLICACIONES ÉTICAS DEL PROYECTO A DESTACAR

RIESGOS DE LA INVESTIGACIÓN Y BENEFICIOS POTENCIALES:

El presente proyecto seguirá los principios éticos y deontológicos relativos a la investigación psicológica recogidos en los principales códigos actualmente existentes a nivel nacional o internacional: El Código del Colegio Oficial de Psicólogos (1987), el Meta-Código de la Federación Europea de Asociaciones de Psicólogos (1995), y los Principios Éticos y Código de Conducta de los Psicólogos de la Asociación Americana de Psicología (2002). Se tendrá especial cuidado con respecto al consentimiento informado, la voluntariedad en la participación, y el derecho a abandonar el estudio en cualquier momento.

A tenor de los conocimientos existentes en este campo hasta el momento, no existe nada en el proyecto que implique riesgos para los/as participantes. El protocolo de evaluación se compone de instrumentos estandarizados y validados que no suponen riesgo para los/as participantes y serán aplicados y supervisados por personal experto.

La coordinadora de este proyecto declara que: la investigación propuesta respeta los principales fundamentales de la Declaración de Helsinki, del Convenio del Consejo de Europa relativo a los derechos humanos y la Biomedicina, de la Declaración Universal de la UNESCO sobre el genoma humano y los derechos humanos, y del Convenio para la protección de los derechos humanos y la dignidad del ser humano con respecto a las aplicaciones de la Biología y la Medicina.

Los/as investigadores/as del equipo conocen y cumplirán la legislación vigente y otras normas reguladoras, pertinentes al proyecto, en materia de ética, y experimentación con humanos.

Respecto a los beneficios potenciales, la información recabada en este estudio será relevante en tanto en cuanto nos ayude a realizar posibles mejoras en la aplicación, tanto en su contenido como en su diseño, y nos guíe para el futuro desarrollo de un estudio piloto. Además, los participantes podrán beneficiarse gratuitamente de todos los contenidos de la aplicación, los cuales están diseñados para mejorar el bienestar psicológico.

INFORMACIÓN Y CONSENTIMIENTO:

Los participantes recibirán un documento de consentimiento informado que deberán firmar después de recibir una explicación general del procedimiento. En el consentimiento se informará a los participantes de que su participación en el estudio es voluntaria y que pueden decidir no participar y retirar el consentimiento en cualquier momento.

PROTECCIÓN Y TRATAMIENTO DE LOS DATOS/CONFIDENCIALIDAD:

Los datos relativos a los cuestionarios serán recabados mediante la plataforma *Limesurvey* (https://encuestas.uv.es) y almacenados posteriormente en *Núvol* (https://nuvol.uv.es) con la cuenta de la UV de la investigadora principal del presente estudio (banos@uv.es). La responsable de protección de datos (de custodiar los datos del proyecto y del consentimiento informado) también será la investigadora principal del proyecto:

Rosa María Baños Rivera
Facultad de Psicología. Avda. Blasco Ibáñez, 21, 46010. Valencia.
Teléfono: 96 162 54 12
banos@uv.es

Para la identificación de los participantes en los cuestionarios de evaluación y en el proceso de análisis de datos se utilizará un código numérico (nunca su nombre). La información personal será almacenada en una base separada con los códigos numéricos correspondientes y tan solo los miembros del equipo investigador tendrán acceso a las bases de datos.

Se seguirá en todo momento los requisitos que establece la Ley Orgánica de Protección de Datos (Ley 15/1999 de 13 de diciembre) así como el reglamento (UE) 2016/679 del Parlamento Europeo y del Consejo, de 27 de abril de 2016, Real Decreto – ley 5/2018.
Se seguirá en todo momento los requisitos que establece la Ley Orgánica de Protección de Datos (Ley 15/1999 de 13 de diciembre) así como el reglamento (UE) 2016/679 del Parlamento Europeo y del Consejo, de 27 de abril de 2016, Real Decreto – ley 5/2018, y el reglamento de la República de Paraguay (Ley nº 1682/01 y nº 1969/02).

Firmado: Rosa Mª Baños Rivera

FORM EH-1
REQUEST FOR APPROVAL REPORT FROM HUMANRESEARCH ETHICS COMMITTEE

**RESEARCH PROJECT DATA**
**TITLE:** Digital technology to optimize mental health and well-being in chronic patients during the COVID-19 crisis in Paraguay.
**PROJECT DURATION:** 12 months
**SPECIFY WHETHER OR NOT THE PROJECT HAS BEEN EVALUATED BY ANOTHER COMMITTEE:** No
**PROJECT CHARACTERISTICS**
**JUSTIFICATION OF THE PROJECT:**<br>The coronavirus pandemic, caused by the SARS-CoV-2 virus, has triggered a global health alert and has been spreading rapidly around the world since its emergence. According to WHO, mortality caused by infectious diseases, such as COVID-19, occurs more frequently in vulnerable at-risk populations, including the elderly or patients with previous chronic diseases (COPD, cancer, diabetes, heart disease, etc.). In Paraguay, according to the official website of the Ministry of Public Health and Social Welfare, 174 cases were recorded on April 15, with 8 deaths and 30 recovered, after which the government implemented prevention measures, intervention and mitigation strategies (suspension of mass events, exceptional working hours,

nighttime mobility restrictions, border closures, etc.), which has led Paraguay to a situation of strict containment.

The COVID-19 pandemic and the measures taken to control its spread (confinement, cessation of activities, limitation of movements) are having an enormous impact at the health, social and economic level, but also psychologically. Recently published studies (Brooks et al., 2020; Duan & Duan, 2020; Montemuro, 2020) point out that these measures, together with the situation generated by the pandemic (risk of getting sick, possibility of losing one's job,...) will have a negative impact on mental health, increasing the rates of stress and anxiety, as well as other psychological problems (irritability, depression,...) in the general population, and more especially, in vulnerable and/or at risk people (over 60 years old, chronically ill, health care workers,...). Moreover, these problems are expected to be exacerbated, and if these negative effects are not treated early, they could be prolonged even after the end of the quarantine and the pandemic. Therefore, it is necessary to have effective psychological tools to combat the psychological and emotional problems generated by the outbreak of COVID-19, targeting particularly vulnerable groups, such as patients with chronic diseases.

So far, studies conducted on the effects of the pandemic and confinement on mental health indicate that this situation significantly increases stress levels, producing an increase in anxiety, depression, loneliness, etc., with negative consequences that can lead to the development of mental disorders (Brooks et al., 2020; Duan & Duan, 2020; Montemuro, 2020). In addition, the constant news about the virus, together with the restrictive measures to be taken to reduce its contagion rate, entail great emotional stress for the entire population, but especially for at-risk groups, such as people with chronic disease, in whom their chronic condition may even be aggravated, resulting in a worse management of their chronic disease and collapse the health services unnecessarily if they go to them at any apparent symptom. Therefore, reinforcement and behavioral guidance is needed to enable people with chronic diseases to know and understand the necessary measures to prevent infection, or to act if they are infected, while at the same time it is necessary to offer these people tools to help them prevent psychological and emotional problems that can worsen their medical situation and their quality of life.

Studies on the establishment and maintenance of new health behaviors show that it is not enough to provide the necessary information to the patient. This information is a necessary and fundamental component, but it is not sufficient to bring about this behavioral change. The theories developed to study human behavior postulate the existence of different phases and factors (sociodemographic, cultural, social, etc.) for effective behavioral change to occur. Within the models, the Integrated Behavior Change (I-Change) model (De Vries, 2017) integrates the contributions of models such as: a) the Attitude - Social Influence - Self-Efficacy model; b) the Theory of Planned Behavior; c) the Social Cognitive Theory; d) goal setting; e) the Health-Belief model; and f) the Transtheoretical Behavior Change model (De Vries, 2018; De Vries, et al., 2018; De Vries, Mudde, & Dikstra, 2000). The I-Change model proposes that behavior change has four steps*: awareness, motivation, action, and acquired behavior*. People move from the first to the fourth based on different determinants of behavior at each stage, which are conditioned by information externalities (channel, source, message, and interlocutor) that can be modified, and internal factors (biological, psychological, behavioral, environmental) that cannot be modified.

Therefore, behavioral and emotional education and empowerment of chronic patients at risk of infection is essential, as relevant information and appropriate and effective strategies can facilitate patients to engage in specific actions (health behaviors) and behavioral, attitudinal and

emotional changes (e.g., personal hygiene measures, adherence to therapeutic prescriptions, etc.) that result in better health management and quality of life. In addition, teaching and training in effective psychological strategies and interventions can help these patients to more effectively manage their emotional distress and improve their quality of life.

Following the outbreak of COVID-19, digital tools have become essential to detect, contain and seek treatment and support for the disease. These tools present a number of advantages over traditional face-to-face treatments, such as: a) accessibility; b) flexible use anytime, anywhere; c) adaptability to the user's pace; d) anonymity; e) standardized content, allowing progress without the presence of the therapist; f) content tailored to the target population; and f) low costs in the provision of mental health services (Schröder et al., 2016). As the recent article published in the prestigious journal JMIR (Torous et al, 2020) points out, the pandemic has made the potential of digital health to increase access and quality of mental health services even more evident, and it can be argued that at this time "telehealth" solutions are the right and appropriate solution to provide mental health care in the current crisis. Mental health applications represent an opportunity to expand access and quality of psychological treatments (Anthes, 2016). Different organizations and institutions related to mental health care point out that this type of technology can provide cost-effective and scalable solutions to the current treatment gap (East and Havard, 2015). Specifically, mobile apps can be a useful tool for delivering psychological treatments, compared to other platforms, due to: a) ease of habit, b) low effort expectation, and c) high hedonic motivation (East & Havard, 2015; Yuan et al., 2015). In addition, mental health apps can span all stages of clinical care delivery, from prevention to post-treatment condition management (i.e., relapse prevention) (Price et al., 2014).

Therefore, solutions using mobile technologies made through a secure channel of action, with a high level of penetration in the population, scalable, and with a relatively low cost, make them perfect as a tool to carry out public health strategies in this area. In addition, the lack of specific digital solutions for educating and empowering chronic patients in the prevention of infectious diseases such as PCVID-19 and other pandemic diseases means that these patients have to visit health centers and hospitals frequently, increasing the risk of infection. As these are rapidly transmitted diseases, prevention plays an important role and transmission of viruses presents special risks within healthcare facilities and can cause explosive outbreaks of disease causing serious problems for the healthcare system.

This research project proposes the use of technologies to provide a psychological support program specifically aimed at a particularly vulnerable population during the pandemic: the chronically ill. The program, called "Mejora.CareTM", has been developed by Saludmedia Labs in Spain and is part of the AdheraHealthTM platform. This platform has shown its effectiveness as a tool to offer emotional support and promote behavioral changes in different clinical contexts (Carrasco et al., 2020; Luna-Perejon et al., 2019; Hors-Fraile et al., 2019).

Mejora.CareTM is aimed at offering support to patients with chronic diseases. On the one hand, educational information will be provided to help them to better understand their illness, to follow medical indications and to improve their infection prevention behaviors. On the other hand, tools for self-care and emotional management of stress, anxiety and other negative emotions generated by the pandemic crisis and its consequences will be provided, especially in the target population of this study, since it is considered a high-risk population. In this sense, the proposed technology will raise awareness, educate and empower chronic patients, encouraging behavioral changes that promote self-care to reduce the risks of COVID-19 infection. At the

same time, it will offer emotional management tools with the aim of improving their mental health, quality of life and emotional well-being.

Mejora.CareTM offers patients the following components:
(a) symptom monitoring; (b) patient empowerment components (including educational methods, training exercises for anxiety and negative emotion management, and instructions to promote behavioral changes to institute and/or maintain a healthy lifestyle that minimizes the risk of contagion/propagation of the disease and improves patients' quality of life); (c) access to related information issued from official sources; and (d) access to healthcare resource mapping.

The program also offers tools to healthcare providers, such as:
-The segmentation and follow-up of potential COVID-19 patients and high-risk individuals: including follow-up tools through messages that can be customized to the needs of a particular patient or a specific group.
- Support for patients in the home recovery phase: including tools for real-time monitoring of symptoms, follow-up of worsening or improvement, communication through messages to provide support, advice or personalized instructions.

In summary, this technological tool will allow the collection of real data on the level of knowledge, behaviors, and symptoms, which will enable analysis and improve decision making by health authorities. The use of this digital technology is expected to have a great impact on the quality of life of patients with chronic diseases, as well as an improvement in the optimization of the services offered by the public health system. The platform is also expected to contribute to raising awareness and educating patients, allowing them to be assisted and offered emotional support without the need to attend health centers and hospitals.

References

Andersson, G. & Titov, N. (2014). Advantages and limitations of Internet-based interventions for common mental disorders. *World Psychiatry, 13*(1), pp.4-11.

Andrews, G., Basu, A., Cuijpers, P., Craske, M.G., McEvoy, P., English, C.L., & Newby JM. (2018). Computer therapy for the anxiety and depression disorders is effective, acceptable and practical health care: an updated meta-analysis. *Journal of Anxiety Disorders, 55*, 70-78.

Anthes, E. (2016). Mental health: There's an app for that. *Nature, 532*(7597), 20-23.

Botella, C., Quero, S., Baños, R. & García-Palacios, A. (2009). Avances en los tratamientos psicológicos: la utilización de las nuevas tecnologías de la información y la comunicación. *Anuario de psicología/The UB Journal of Psychology, 40*(2), pp.155-170.

Brooks, S.K., Webster, R.K., Smith, L.E., Woodland, L., Wessely, S., Greenberg, N., & Rubin, G.J. (2020). The psychological impact of quarantine and how to reduce it: rapid review of the evidence. *The Lancet, 395*(10227). https://doi.org/10.1016/S0140-6736(20)30460-8

Carrasco-Hernández, L., Jódar-Sánchez, F., Nuñez-Benjumea, F., Moreno-Conde, J., Mesa-González, M., Civit-Balcells, A., Hors-Fraile, S., … Ortega-Ruiz, F.A. (2020) Digital Therapeutics Solution Complementing Psychopharmacology-Supported Smoking Cessation: Randomized Controlled Trial. *JMIR mHealth*. DOI: 10.2196/17530

De Vries, H. (1998). Planning and evaluating health promotion. Evaluating health promotion.

De Vries, H. (2017). An integrated approach for understanding health behavior: The I-Change Model as an example. *Psychology and Behavioral Science International Journal,* 2(2).

De Vries, H., et al., (1998). Differential beliefs, perceived social influences, and self-efficacy expectations among smokers in various motivational phases. *Preventive Medicine*, *27*(5): p. 681-689.

De Vries, H., Mudde, A.& A. Dijkstra (2000). The attitude-social influence-efficacy model applied to the prediction of motivational transitions in the process of smoking cessation. Understanding and changing health behaviour: From health beliefs to self-regulation, p. 165-187.

Duan, L., & Zhu, G. (2020) Psychological interventions for people affected by the COVID-19 epidemic. *Lancet Psychiatry. 7*(4), 300-302.

East, M.L., & Havard, B.C. (2015). Mental health mobile apps: from infusion to diffusion in the mental health social system. *JMIR Mental Health, 2(*1), e10.

Giwa, A.L., Desai, A., & Duca, A. (2020) Novel 2019 Coronavirus SARS-CoV-2 (COVID-19): An Updated Overview for Emergency Clinicians. *Emerg Med Pract; 21(*5): 1-28.

Hors-Fraile. S., Vries, H., Malwade, S., Luna-Perejon, F., Amaya, C., Civit, A., ... & Li, Y. C. (2019) Opening the Black Box: Explaining the Process of Basing a Health Recommender System on the I-Change Behavioral Change Model, in IEEE Access, vol. 7, pp. 176525-176540. doi: 10.1109/ACCESS.2019.2957696

Kazdin, A.E. & Blase, S.L. (2011). Rebooting psychotherapy research and practice to reduce the burden of mental illness. *Perspectives on Psychological Science, 6*(1), pp.21-37.

Kazdin, A.E. & Rabbitt, S.M. (2013). Novel models for delivering mental health services and reducing the burdens of mental illness. Clinical Psychological Science, 1(2), pp. 170-191.

Luna-Perejon, F., Malwade, S., Styliadis, C., Civit, J., Cascado-Caballero, D., Konstantinidis, E., ...Li, Y.J. (2019) Evaluation of user satisfaction and usability of a mobile app for smoking cessation. *Comput Methods Programs Biomed, 182*:105042. doi: 10.1016/j.cmpb.2019.105042

Ministerio de Salud Pública y Bienestar Social, Dirección General de Vigilancia de la Salud. Boletín de Vigilancia N3 Enfermedades No Transmisibles y Factores de Riesgo, 2019. Asunción.

Ministerio de Salud Pública y Bienestar Social, Dirección General de Vigilancia de la Salud, 2017. Política Nacional de Calidad en Salud 2017-2030. ISBN 978-99967-36-61-2

Montemurro N. (2020) The emotional impact of COVID-19: From medical staff to common people. *Brain Behav Immun*, S0889-1591(20) 30411-6. doi:10.1016/j.bbi.2020.03.032

Organización Mundial de la Salud. (2020) Statement on the second meeting of the International Health Regulations (2005) Emergency Committee regarding the outbreak of novel coronavirus (2019-nCoV). Nota de prensa. Disponible en: https://www.who.int/news-room/detail/30-01-2020-statement-on-the-second-meeting-of-the-international-health-regulations-(-005)-emergency-committee-regarding-the-outbreak-of-novel-coronavirus-(2019-ncov)

Price, M., Yuen, E. K., Goetter, E. M., Herbert, J.D., Forman, E.M., Acierno, R., & Ruggiero K.J. (2014). mHealth: a mechanism to deliver more accessible, more effective mental health care. *Clinical Psychology & Psychotherapy, 21*(5),427-436.

Schröder, J., Berger, T., Westermann, S., Klein, J. P., & Moritz, S. (2016). Internet interventions for depression: new developments. *Dialogues in Clinical Neuroscience, 18*(2), 203-212.

Torous, J., Jän Myrick, K., Rauseo-Ricupero, N., & Firth, J. (2020) Digital Mental Health and COVID-19: Using Technology Today to Accelerate the Curve on Access and Quality Tomorrow. *JMIR Ment Health, 7*(3):e18848. doi:10.2196/18848

Yuan, S., Ma, W., Kanthawala, S. & Peng, W. (2015). Keep using my health apps: discover users'perception of health and fitness apps with the UTAUT2 model. *Telemedicine and e-Health, 21*(9), 735-741.

RESEARCH OBJECTIVES:

The **main objective** of this project is to study the effects of an intervention (Mejora.CareTM) aimed at providing support and information, and training empowerment skills in patients with chronic diseases (COPD, cancer, diabetes, heart disease. hypertension, etc.) during the COVID-19 crisis.<br><br>This objective is broken down into the following **specific objectives**:<br>1. To evaluate the effectiveness of the use of the Mejora.CareTM tool in improving the levels of quality of life and the reduction of anxiety and depression symptomatology in patients with chronic diseases compared to a control group.<br>2. To evaluate the acceptance of the Mejora.CareTM tool by the users.<br><br>The **research hypotheses** are as follows:<br><br>**Hypothesis 1**. Patients using the application are expected to have increased levels of quality of life and emotional well-being compared to the control group at 1-, 3-, 6-, and 12-month follow-ups.<br><br>**Hypothesis 2**. Patients using the application are expected to have a significant reduction in levels of anxiety and depression compared to the control group at the 1-, 3-, 6-, and 12-month follow-ups.<br><br>**Hypothesis 3**. Patients will positively value the usefulness of the application and will show high levels of satisfaction and acceptance.
**METHODOLOGICAL DESIGN:** The design of this project is a randomised controlled trial with two arms (intervention group and control group). The study will be conducted with chronic patients belonging to hospitals in the Itapúa region and will be conducted following the CONSORT (Consolidated Standards of Reporting Trials, http://www.consort-statement.org) (Moher et al., 2010), CONSORT-EHEALTH (Eysenbach, 2011) guidelines and the Recommendations for Interventional Trials (SPIRIT).
**PLACE OF RESEARCH:** Hospitals in the Itapúa region of Paraguay.
**SAMPLE SIZE:**<br>Regarding the required sample size, using the G-Power program (Faul et al., 2007), it has been established that 39 participants per condition (78 in total) are necessary, taking into account a moderate expected effect size on the main measure of change (f = 0.25) according to Liu et al. (2011) and Ramachandran et al. (2007), a statistical power of .80, and an alpha of .05. Anticipating a possible dropout of 20% the total minimum sample size will amount to 94 patients.
**SELECTION CRITERIA FOR PARTICIPANTS:**<br>Inclusion criteria will be: a) be over 18 years of age; b) ability to understand and read Spanish; c) have a smartphone-type cell phone with Internet connection; d) be diagnosed with a chronic disease (i.e., COPD, cancer, diabetes, heart disease, hypertension); e) provide written informed consent.<br><br>Exclusion criteria will be: a) lack of technological knowledge and inability to use the cell phone; b) diagnosis of psychiatric disorders or severe cognitive impairment.

COLLECTED VARIABLES IN THE RESEARCH AND DESCRIPTION OF THE INTERVENTIONS TO BE CARRIED OUT:

Measures:

Sociodemographic variables and medical history
Personal data will be collected, including information such as age, gender, profession, marital status, employment status, level of studies, diagnosis of chronic disorder and its duration, clinical course, medication, diagnosis of mental disorder, and pregnancy (Baseline).

User's technological profile
- The Computer Fluency Scale (CFS; Becker, 2012) (Baseline).
- *Ad hoc* questionnaire designed to evaluate the frequency and the perceived ability to use a mobile phone (Baseline).

Primary outcome measure
- **Quality of life:** The EuroQol 5-Dimensions 3-Levels Questionnaire (EQ-5D-3L; Badia et al., 1999; EuroQol Group, 1999) (Baseline, 1, and 3, 6 and 12 months).

Secondary outcome measures
- **Anxiety symptoms:** The Generalised Anxiety Disorder Questionnaire-2 (GAD-2; Kroenke et al., 2007; García-Campayo et al., 2012) (Baseline, 1, and 3, 6 and 12 months).
- **Depressive symptoms:**The Patient Health Questionnaire-2 (PHQ-2; Kroenke et al., 2003; Rodríguez-Muñoz et al., 2017) (Baseline, 1, and 3, 6 and 12 months).
- **Stress:** The Perceived Stress Scale-4 (PSS-4; Herrero et al., 2006; Vallejo et al., 2018) (Baseline, 1, and 3, 6 and 12 months).
- **Self-efficacy**: The General Self-Efficacy Scale-12 (GSES-12; Bosscher et al., 1997; Herrero et al., 2014) (Baseline, 1, and 3, 6 and 12 months).
- **Health empowerment**: The Health Empowerment Scale (HES; Serrani et al., 2014) (Baseline, 1, and 3, 6 and 12 months).
- **System usability**: The System Usability Scale (SUS; Brooke, 1996; Sevilla-Gonzalez et al., 2020) (1 month)
- **Telehealth usability**: An adaptation of the original Telehealth Usability Questionnaire (TUQ; Parmanto et al., 2016; Torre et al., 2020) (1 month).
- **App usability:** The mHealth App Usability Questionnaire (MAUQ; Attkisson et al., 1996) (1 month).
- **Client satisfaction**: The Client Satisfaction Questionnaire (CSQ; Larsen et al., 1979; Roberts et al., 1984) (1 month).

Interventions:

Mejora.CareTM

Participants assigned to the intervention group will access the Mejora.CareTM application through their mobile device or Smartphone. This mobile application includes the following functionalities: a) symptom monitoring; b) patient empowerment components (including educational methods, training exercises for managing anxiety and negative emotions, and instructions to promote behavioral changes to establish and/or maintain a healthy lifestyle that

minimizes the risk of contagion/propagation of the disease and improves patients' quality of life); c) access to related information issued from official sources; and d) access to a map of healthcare resources. The application will have the following contents:

- Easy-to-understand educational content about the new coronavirus and how to prevent contagion, as well as useful information for managing emotions in this situation. As part of the learning program, the contents include self-assessment tests that allow to check and reinforce the acquired knowledge.
- Automated personalized messages that help people to acquire lifestyle habits to prevent infection by the coronavirus. The contents of the messages have been validated by health professionals, experts in behavior change and communication and health promotion.

Control group: Waiting list

The WL group will not receive any intervention during the 1-year study period.

Data analysis:

Descriptive statistics, t-tests for independent samples, ANOVAs, and chi-square analysis will be calculated to observe differences between groups at baseline in quantitative and categorical variables. Linear mixed models will be performed for each dependent variable, considering temporal moments as an intra-group factor and condition as an inter-group factor. Bonferroni correction will be used for posthoc multiple comparisons, and Cohen's d effect size will be calculated for intra- and inter-group comparison. Multiple hierarchical regression models will be used to identify possible predictors of evolution in the primary and secondary measures. Finally, mediation and moderation analyses will be performed to analyse the variables responsible for the intervention's effect on the dependent variables and to detect the profile of patients who will benefit most from the intervention.

ETHICAL IMPLICATIONS OF THE PROJECT

RESEARCH RISKS AND POTENTIAL BENEFITS:

The present project will follow the ethical and deontological principles related to psychological research contained in the main codes currently existing at the national or international level: The Code of the Official College of Psychologists (1987), the Meta-Code of the European Federation of Psychologists' Associations (1995), and the Ethical Principles and Code of Conduct for Psychologists of the American Psychological Association (2002). Special care will be taken with respect to informed consent, voluntariness of participation, and the right to leave the study at any time.

Based on existing knowledge in this field to date, there is nothing in the project that poses any risk to participants. The evaluation protocol consists of standardized and validated instruments that do not pose any risk to the participants and will be applied and supervised by expert personnel.

The coordinator of this project declares that: the proposed research respects the fundamental principles of the Declaration of Helsinki, the Council of Europe Convention on Human Rights and Biomedicine, the UNESCO Universal Declaration on the Human Genome and Human

Rights, and the Convention for the Protection of Human Rights and Dignity of the Human Being with regard to the Application of Biology and Medicine.

The researchers of the team are aware of and will comply with current legislation and other regulatory norms relevant to the project regarding ethics and human experimentation.

Regarding the potential benefits, the information gathered in this study will be relevant insofar as it will help us to make possible improvements in the application, both in its content and design, and will guide us for the future development of a RCT. In addition, participants will be able to benefit free of charge from all the contents of the application, which are designed to improve psychological well-being.

INFORMATION AND CONSENT:

Participants will receive an informed consent document that they will be asked to sign after receiving a general explanation of the procedure. The consent will inform participants that their participation in the study is voluntary and that they can decide not to participate and withdraw consent at any time.

DATA PROTECTION AND DATA PROCESSING/CONFIDENTIALITY:

Data related to the questionnaires will be collected through the Limesurvey platform (https://encuestas.uv.es) and subsequently stored in Núvol (https://nuvol.uv.es) under the UV account of the principal investigator of the present study (banos@uv.es). The person responsible for data protection (for safeguarding the project data and informed consent) will also be the principal investigator of the project:

Rosa María Baños Rivera
Facultad de Psicología. Avda. Blasco Ibáñez, 21, 46010. Valencia.
Teléfono: 96 162 54 12
banos@uv.es

For the identification of participants in the evaluation questionnaires and in the data analysis process, a numerical code will be used (never their name). Personal information will be stored in a separate database with the corresponding numerical codes and only members of the research team will have access to the databases.

The requirements established by the Organic Law on Data Protection (Law 15/1999 of December 13) as well as the regulation (EU) 2016/679 of the European Parliament and of the Council of 27 April 2016, Royal Decree - law 5/2018 will be followed at all times.

The requirements established by the Organic Law on Data Protection (Law 15/1999 of December 13) as well as the Regulation (EU) 2016/679 of the European Parliament and of the Council of April 27, 2016, Royal Decree - law 5/2018, and the regulation of the Republic of Paraguay (Law No. 1682/01 and No. 1969/02) will be followed all the time.

Signed: Rosa Mª Baños Rivera
